# Impedance-based tracking of a cochlear implant electrode reliably detects successful array insertion

**DOI:** 10.1007/s00405-026-10464-w

**Published:** 2026-09-16

**Authors:** Tabita Breitsprecher, Stefan Hans, Aline Föger, Angelika Dierker, Joachim Schmutzhard, Stefan Dazert, Nora M. Weiss

**Affiliations:** 1 University Medical Center Hamburg-Eppendorf, Hamburg, Germany; 2 Department of Otorhinolaryngology-Head and Neck Surgery, Ruhr-University Bochum, St. Elisabeth-Hospital Bochum, Bochum, Germany; 3 MED-EL, Elektromedizinische Geräte Deutschland GmbH, Starnberg, Germany; 4 MED-EL, Elektromedizinische Geräte GmbH, Innsbruck, Austria; 5 Department of Otorhinolaryngology, Medical University, Innsbruck, Austria; 6 Klinikum Rechts Der Isar, Department of Otorhinolaryngology, TUM School of Medicine, Munich, Germany

**Keywords:** Impedance telemetry, Feedback mechanism, Insertion monitoring, Anatomy-based fitting

## Abstract

**Introduction:**

Continuous impedance measurements during cochlear implantation have been hypothesized to provide a reliable indication if the electrode is inside or outside the cochlea. Therefore they provide a valuable feedback mechanism for successful insertion to the surgeon. This study aims to investigate whether impedances can be used to reliably detect successful insertion of the cochlear -implant electrode array.

**Methods:**

In a prospective cross-sectional single-center study patients undergoing cochlear implantation with flexible lateral wall arrays (MED-EL, Innsbruck, Austria) were recruited between June 2022 and May 2023. Continuous impedance telemetry during electrode array insertion was obtained and compared to the ground truth of visual and audiological feedback by the surgeon.

**Results:**

34 patients (23 women, 11 men;, 16 left ears, 18 right ears) with a mean age of 52.8 years (SD 19.6 years) were included. The present study found a reliability of 88.9% comparing the algorithm of continuous insertion to the ground truth.

**Conclusions:**

Continuous impedance measurements show a high reliability to detect the insertion of electrode contacts of a cochlear implant electrode array. The data collected by the software are suitable to detect misplacements already during insertion. This method is a promising approach to intraoperatively monitor successful insertion and offers the opportunity to replace fluoroscopy and radiological imaging in the aftercare of cochlear implant surgery.

## Introduction

Cochlear implantation is a standard procedure in cases of severe sensorineural hearing loss or deafness to rehabilitate hearing by electrically stimulating the spiral ganglion cells in the inner ear [1]. The availability of different electrode array types and lengths enables individual selection of the electrode array adapting the insertion depth to the size of the cochlea or the residual hearing. Notably, previous studies showed that preoperative angular insertions depth prediction methods from radiological imaging tend to overestimate the actual insertion depth [2, 3]. Therefore, even if preoperative imaging is indispensable for assessing the anatomical conditions, predictions of the electrode insertion depth based on real-time electrophysiological approaches would be desirable to gain real-time information about the array position. With the advancing development of surgical techniques, electrophysiological measurements have increasingly found their way into the intraoperative monitoring during cochlear implant (CI) surgery [4]. While real-time measurements monitoring structure preservation are already advanced [5–7], electrophysiological methods to objectify the progress of the electrode array insertion are still lacking. Still, the gold standard for monitoring the electrode array position is the postoperative determination of successful insertion using radiographic imaging. This is either required immediately after surgery to prove successful insertion or in the later clinical course to identify electrode migration. Imaging is usually preceded by intraoperative impedance measurement. The measured impedance value is interpreted as the equivalent of the intracochlear resistance and provides information about the integrity of the implant [8]. Furthermore, impedance measurements have been shown to detect different tissue resistances that vary depending on the size of the cochlea [9], the site of stimulation [10], and the amount of fibrous tissue formation [11–14]. Additionally, impedance telemetry and its visualization in the impedance matrix may allow the early detection of anomalous CI electrode position such as tip-fold over, basal kinking, fold-back, or buckling [15] and open the opportunity for immediate intraoperative correction. Previous studies showed promising results that impedance telemetry can even be correlated with angular insertion depth [9, 10].

Since the results from these studies showed differences in impedances depending on the surrounding tissue and the electrode array position inside the cochlea, we aimed to investigate if impedance telemetry provides valid real-time information about intra- or extracochlear positioning of electrode array contacts during the insertion process. Precise real-time feedback providing reliable information about misplacements such as tip-foldovers or extracochlear electrode contacts, has the potential to reduce the need for postoperative radiographic imaging, which is especially of interest in the treatment of children.

In this study, we hypothesize that continuous impedance measurements during cochlear implantation can provide a reliable indication of whether the electrode is inside or outside the cochlea. Therefore, we tested the applicability of an insertion monitoring software and whether it is suitable to gain reliable electrophysiological information about an intra- or extracochlear position of electrode contacts and a precise real-time feedback about the time of contact insertion.

## Methods

In this prospective single center study, patients that received a CI between June 2022 and May 2023 due to severe or profound hearing loss were included. MED-EL implants with an electrode array length chosen according to the cochlear duct length (aimed insertion depth: 75%) were used.

### Ethical statement

This study protocol was approved by the local Ethics Committee in accordance with the Declaration of Helsinki (Ruhr-University Bochum: Reg.-No.: 21–7373). Written informed consent was obtained from all participants.

### Study software insertion monitoring tool (IMT)

Study software designed for research purpose was used to perform continuous impedance measurements [16]. The IMT interface shows 12 impedance plots, each depicting one of the 12 electrode contacts. In addition, video recording software (Open Broadcaster Software – OBS) was used to record i) the video of the electrode insertion (attached to the microscope), ii) a video of the IMT user interface including all user interactions, and iii) sound. The video data were recorded to allow a generation of a ground truth for the recorded IMT data as described below.

### Surgery

Using a retroauricular approach, mastoidectomy and posterior tympanotomy were performed in preparation for cochlear implantation. Most electrode arrays were inserted via a round window approach; in 2 cases of revision surgery, insertion was via a cochleostomy. The individual surgical steps and the protocol of study measurements can be found in [16].

### Ground truth

The OBS recordings were analyzed postoperatively by independent observers blinded to the video sound. The surgical approach allowed a confident view of the round window approach or cochleostomy in all recordings. Two observers marked the time of insertion for each electrode contact from 1–12, unaware of the surgeon's feedback. The determined time points were defined as ground truth.

### Statistical analysis

Statistical analyses were performed using Microsoft Excel (version 15.29, Microsoft Corporation), Python (version 3.11), and Prism (version 8, GraphPad Software, Boston, USA). The significance level was set to p < 0.05. Data are presented as mean with standard deviation (SD) and as absolute numbers with percentages.

The inter-rater reliability in the video analysis among different investigators was assessed by calculating Pearson’s correlation coefficient. To assess the degree of concordance, the intra-class coefficient (ICC) was assessed using AI (Perplexity AI, San Francisco, USA, 2026, June 3rd).

## Results

**Fig. 1:**
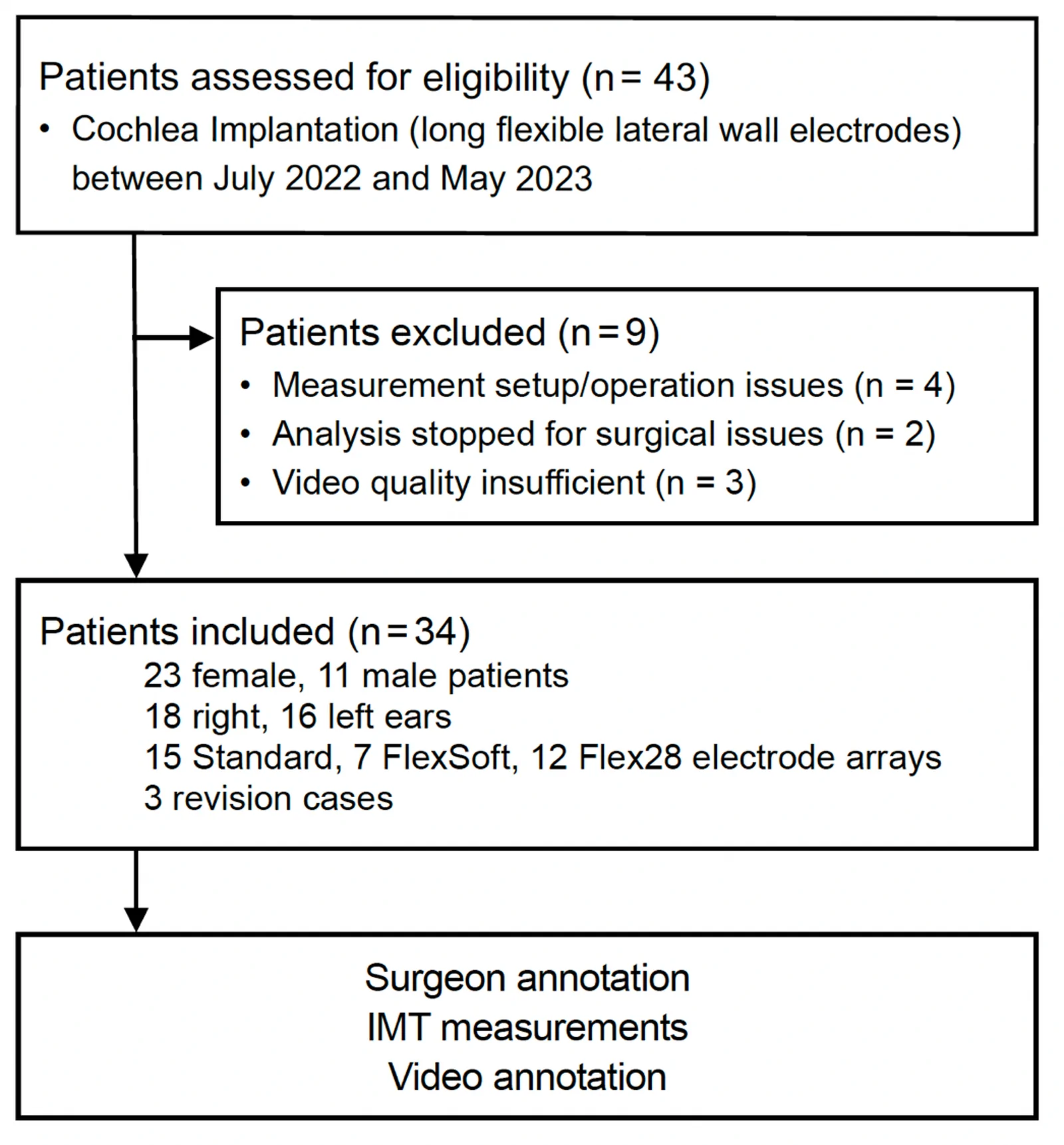
Patient flowchart

Between June 2022 and May 2023, 34 patients (23 women, 11 men; 16 left ears, 18 right ears) with a mean age of 52.8 years (SD 19.6 years) were included in the study. Assessment for patients’ eligibility is shown in Fig. 1.

Of the cases included in this study, 11 28mm electrodes (Flex28) and 23 31.5mm electrodes (16 Standard and 7 FlexSoft) were implanted. Three of the 34 cases were revision cases. In two of the revision cases, a pre-existing cochleostomy was used. In 32 of the cases, a round-window approach was used. The mean insertion speed of electrode contacts 1–12 was 0.44mm per second (SD 0.27mm/s). Only active movement of the array was considered, pauses were subtracted. In all cases, insertion depth of at least 70% was achieved.

In all cases, IMT measurements, surgeon and video annotations were performed and considered valid for further statistical analysis. Pearson’s correlation between the investigators was 0.95 (p < 0.05). The ICC (2,1) was 0.99 corresponding to an excellent inter-rater reliabilty.

**Fig. 2:**
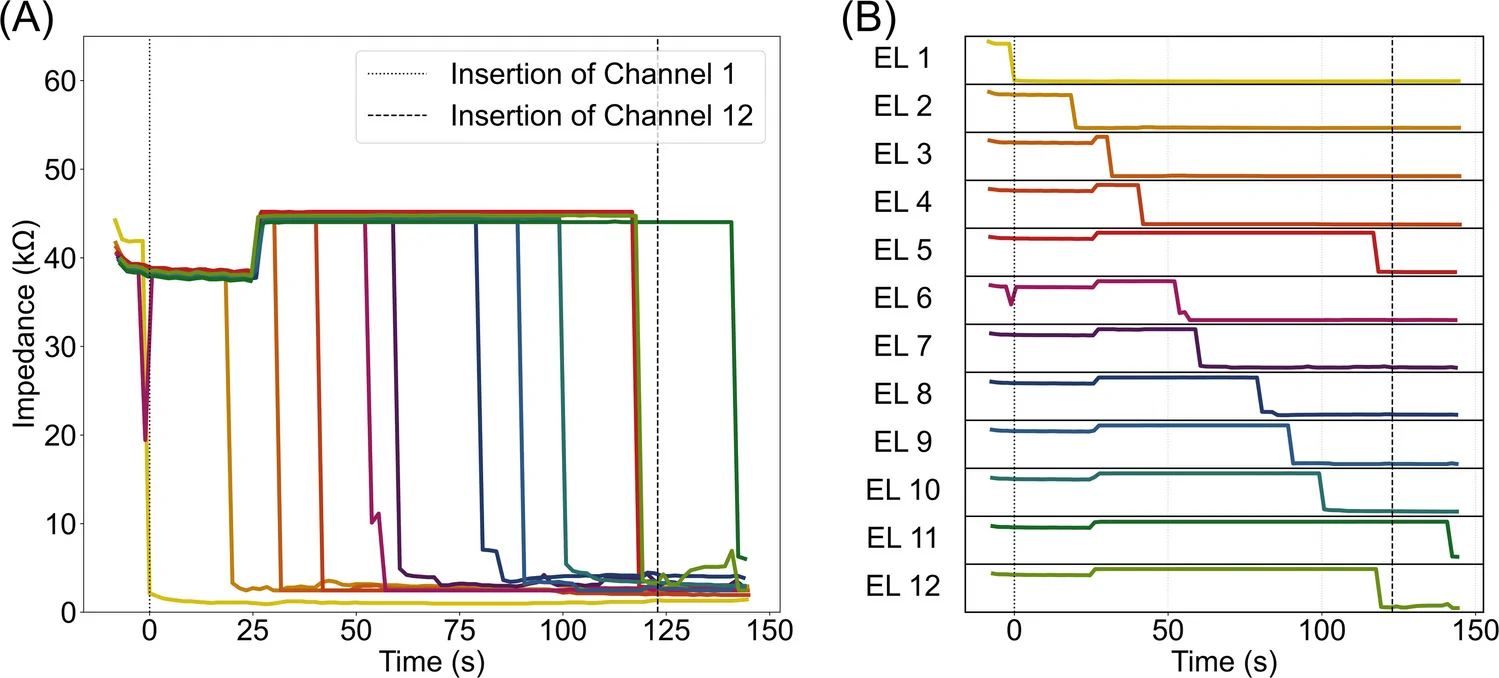
Course of impedances over time for the example of case 37. A: consecutive impedance drops during the insertion of the electrode array. Electrode contacts show immediate impedance drop after insertion. B: Impedance drops resolved for individual electrode contacts, except contact 5. First dashed vertical line corresponds to insertion start, second to end of insertion

Figure 2 shows an example of successful IMT recordings of impedance drops.

**Fig. 3:**
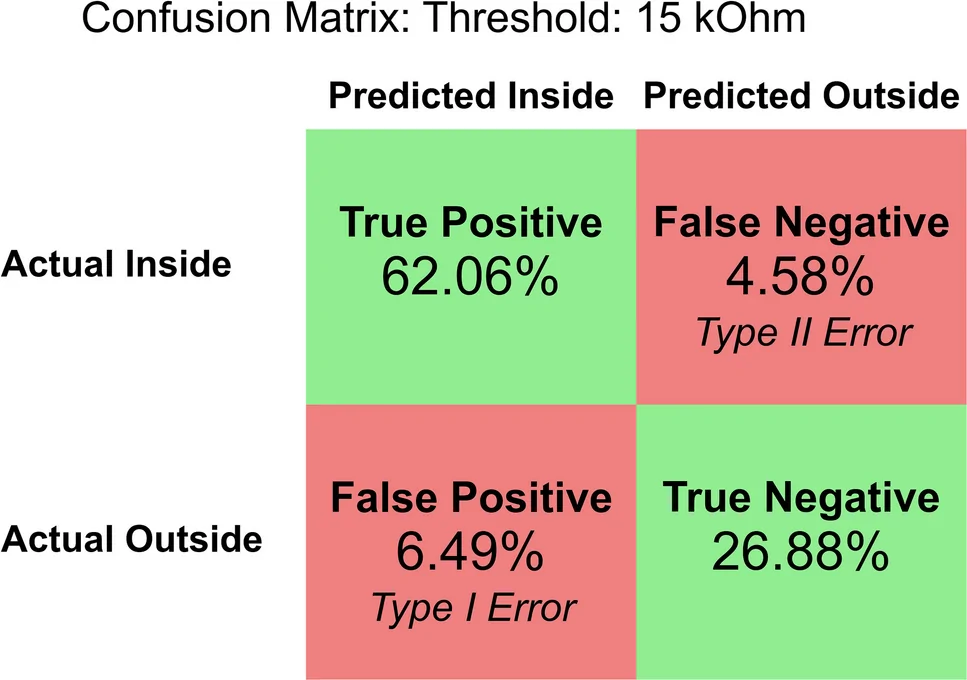
Confusion matrix of error type I and II

In 62.1% of the cases, impedance drops measured by the IMT occurred simultaneously withelectrode contact insertion, corresponding to the ground truth (true positive). In 6.5% of the cases, the IMT detected low impedances when the electrode contact was outside the cochlea (false positive). In 26.9% of the cases, the impedances measured by the IMT were high, in line with electrode contacts being outside the cochlea (true negative). In 4.6% of the cases, the impedances measured by the IMT were high, but electrode contacts were already inserted (false negative, Fig. 3).

We assume that the false positives resulted from premature impedance drops caused by electrode contact with conductive fluid (e.g., perilymph or irrigation fluid) in the round window niche before intracochlear insertion

**Fig. 4:**
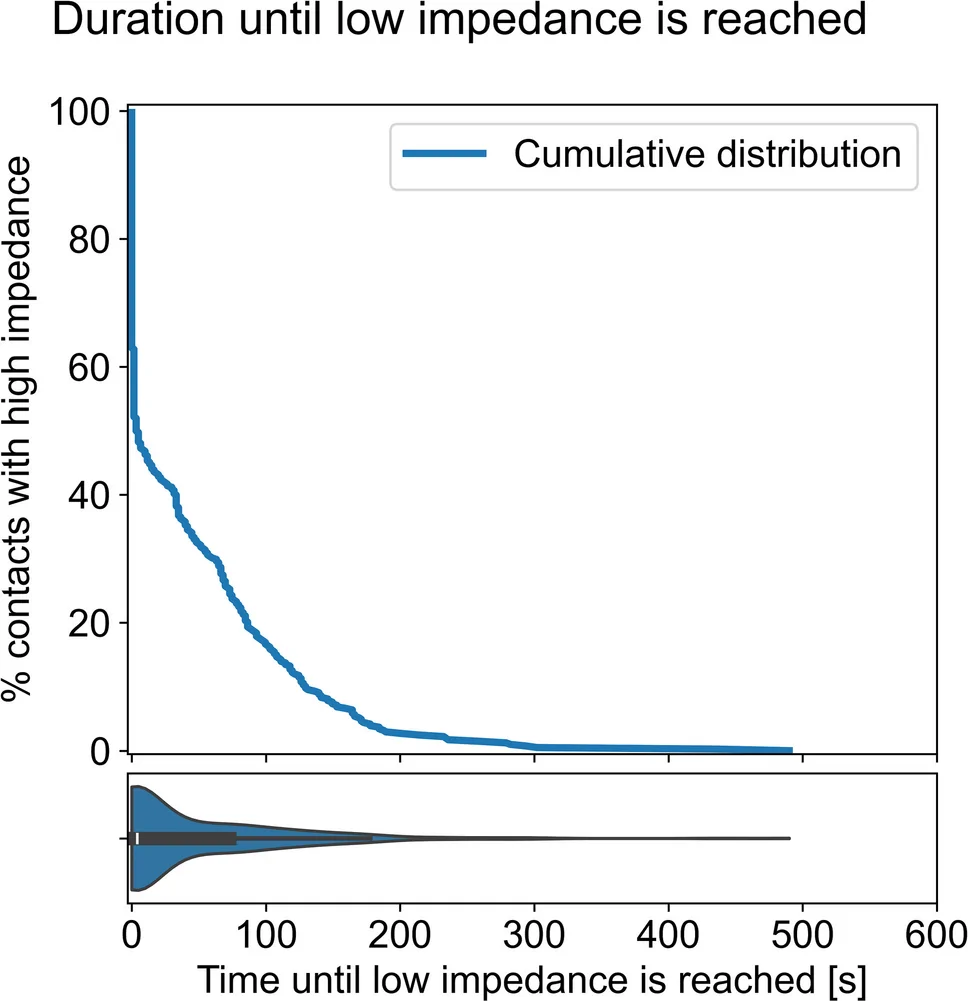
The cumulative distribution in the upper plot shows the percentage of contacts with high impedance over time. For each contact, the time between the insertion of the specific contact until low impedance is reached is computed individually. Data are then sorted by duration. Finally, the percentages of contacts which remain at high impedance are plotted for each time point (starting with 0 at the time of insertion of the specific contact). The curve stops at the point where all contacts achieve low impedance. Overall, low impedance was achieved in 4.15 s (median)

For the type II error, a possible explanation could be high impedance due to air bubbles. In this case, we hypothesized that these high impedances should decrease over time as the air bubbles dissolve. Consequently, we analyzed the cases of type II error for impedance drops over time. Figure 4 summarizes the impedance characteristics for high impedance electrodes over time and shows that all contacts reached low impedance during the measurement time. This supports our hypothesis that all electrode contacts show an impedance drop at some point during surgery.

Due to the high rate of true predictions in 88.9%, we conclude that monitoring impedance data during and shortly after the electrode array insertion is useful to gain real-time information about the intracochlear positioning and may even contribute to detecting dislocations or misplacement (e.g., kinking, buckling, or tip fold-over). To prove this assumption, a case in which an array displacement was detected from postoperative CT was analyzed in more detail.

The evaluation of intraoperative raw data from the IMT is challenging, because there is no visualization of deviations from the norm. Consequently, color-coded voltage heatmaps were created from the intraoperative impedance data retrospectively. The voltage matrices shown below were created retrospectively and were not yet available to the surgeon at the time of insertion in this study.

The voltage heat map is generated by using all 12 electrode channels each serving sequentially as both stimulating and recording channel. All possible combinations were measured which results in a 12 × 12 matrix where the corresponding measure for each stimulation combination was entered. In the resulting matrix, the rows correspond to the stimulating channels and the columns correspond to the recording channels. The heat map illustrates the spatial distribution of the electric field within the cochlea. The stimulus consisted of a predefined burst applied across all contacts, allowing impedance values to be derived from the measured voltages. The impedance in kOhm can be calculated from the voltage values by dividing the voltage by the stimulating current.

**Fig. 5:**
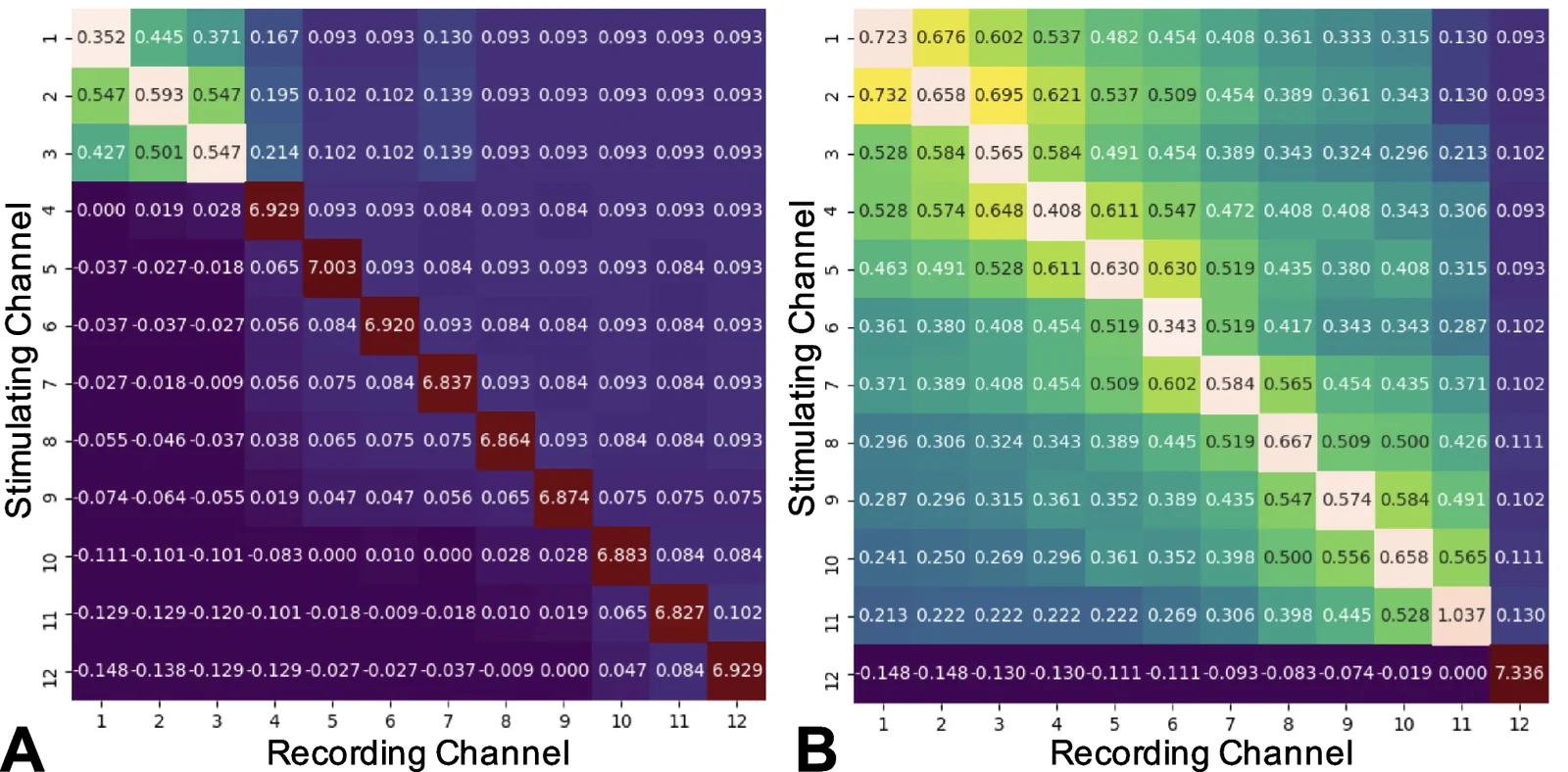
Progressive electrode array insertion with regular voltage field distribution. A: Three electrode contacts inserted recognizable by the pronounced boundary starting from electrode 3, at the time point where 3 electrode contacts were inserted. B: Eleven contacts inserted following deeper insertion during surgery. Only one electrode remaining outside the cochlea, recognizable by the distinct boundary from blue to dark purple beginning at electrode 11

The general color scheme of the voltage heat map has the following characteristics: Cells of the main diagonals indicate the insertion status of the electrode channels: low voltages (beige) indicate contact to fluid (e.g. inserted) whereas high voltages (colored in red) indicate that the contact is in air (e.g. outside). Cells outside the main diagonal show the color-coded voltage values, from purple/dark-blue (low values; recording electrodes not yet inserted) to green (middle values; uniform voltage field between inserted electrodes) to yellow (higher values; higher voltages between inserted electrodes). Figure 5 shows an example of a regular voltage heat map.

**Fig. 6:**
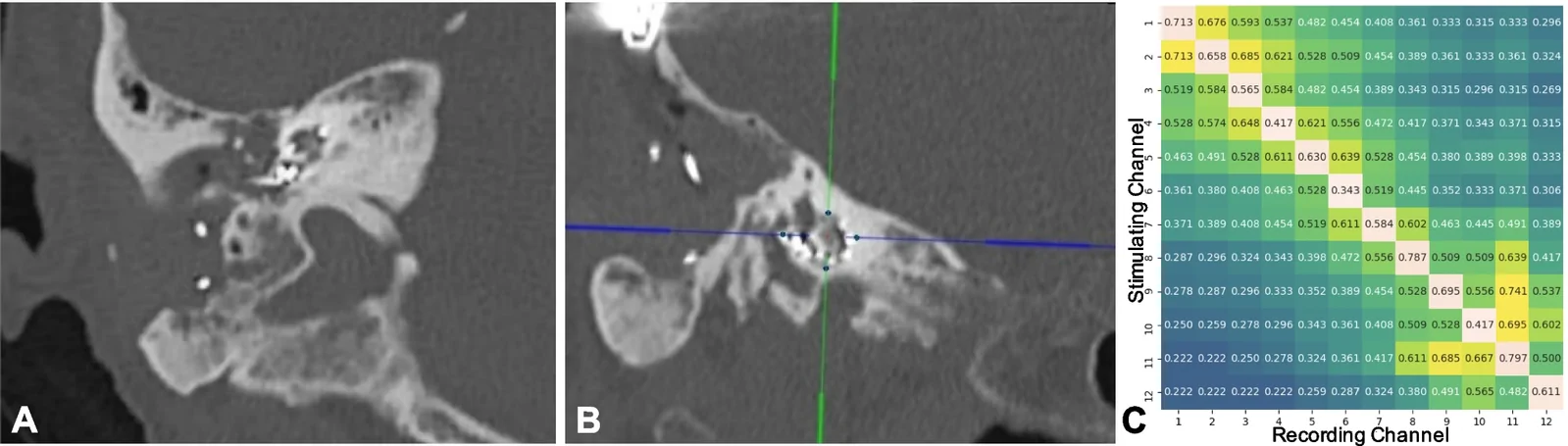
A/B: Postoperative radiograph with complete insertion.Basal kinking/buckling of electrode contacts 11 and 12. C: Heatmap indicating complete electrode insertion, basal buckling visible in contact 11

In one case, postoperative CT imaging revealed a slight basal buckling associated with resistance encountered during insertion. Consequently, postoperatively, the impedance data were analyzed and the heatmap was created (Fig. 6). The heatmap reveals elevated voltage values basally along the diagonal, represented by the yellow coloration.

## Discussion

This study aimed to assess two main aspects: i) the feasibility and reliability of the IMT research software to prove -insertion of individual electrode contacts and complete electrode insertion during CI surgery and ii) compare IMT measurements to a ground truth determined by video analysis. In this study, we found that continuous impedance measurements show a high feasibility and reliability to detect the insertion of electrode contacts of a CI electrode array. The interrater reliability analyzing the ground truth was excellent. In 34 cases IMT setup was possible. This is similar to a recent study by Gottfried et al. [17], reporting a feasibility of 95%. The electrode contacts showed the expected decrease in impedances within 500s. Real-time impedance drop was detected in 62.1% (true positive rate).

We noted that impedances may fluctuate during insertion, e.g., due to pressure changes or air bubbles, but still stabilized by the end of surgery at a low level similar to postoperative values. These possible intraoperative inaccuracies need to be reduced to a minimum for further clinical use. High impedances in our study were interpreted as the occurrence of air bubbles since they decreased during surgery. As a side note, we observed, that high impedances could be reduced with a more constant electrode insertion using an acoustic feedback [18]. Therefore, we recommend the use of an acoustic feedback, especially when using the IMT.

The correlation between impedance telemetry and ground truth was excellent. These findings indicate a high reliability of the research software to detect intra- or extracochlear contact positions. Detecting possible misplacements postoperatively was possible (Fig. 6) from real-time data, especially accompanied by using the voltage matrix. Therefore, we emphasize the development of an intraoperative visualization of impedance matrices. This might be increasingly important in approaches for minimal invasive cochlea implantation such as robotic assisted electrode array insertion [19, 20], in which visual overview is limited compared to conventional approach via mastoidectomy and posterior tympanotomy.

Further development of continuous impedance measurements may even enable the real-time assessment of angular insertion depth. In anatomy-based fitting approaches the audio processor is fit based on information from postoperative radiographic imaging according to the cochlear duct length [21]. As a possible outlook, if real-time impedance measurements reliably reflect electrode position, CI fitting could be guided by intraoperative electrophysiological measurements instead of postoperative radiographic imaging. Therefore, reliable and real-time information about the electrode position intraoperatively appears mandatory for an optimal outcome and further development of these techniques. Thus, we hypothesize that a combination of this knowledge with the ability of continuous stimulation may offer a feedback mechanism for the electrode array position.

### Limitations

Since we suspected changes in impedance behavior and the occurrence of air bubbles might depend on the insertion speed, we introduced a metronome-guided insertion with acoustic feedback every 10s. This was perceived as a helpful tool for slower insertions, and the method was retained for subsequent cases. As these modifications were made during the ongoing study, there is no randomization allowing a statistical analysis of this effect. Eliminating high impedances is the key challenge for the future usability of this method.

Additionally, contact loss between the coil and the implant were considered as a limitation of the continuous impedance measurements. Here, a high standardization concerning the draping and positioning of the surgical instruments is mandatory. In addition, a maximization of the magnet strength in the real time measurement coil could help to minimize this issue.

## Conclusion

Real-time impedance data is valuablefor anatomy-based fitting, in CI recipients with residual hearing, and for reducing the need for postoperative radiographic imaging. Real-time feedback may help to indicate the insertion depth and the correct position of the electrode array without the need for radiographic imaging. The findings from this study demonstrate the feasibility of real-time feedback with continuous impedance measurements during electrode insertion. Nonetheless, further improvement is required to reduce technical limitations such as loss of contact between the implant and coil.
